# Comparing exercise determinants between Black and White older adults with heart failure

**DOI:** 10.1186/s12877-023-04305-2

**Published:** 2023-09-30

**Authors:** Navin Kaushal, Donya Nemati, Dylan Mann-Krzisnik, Adrián Noriega de la Colina

**Affiliations:** 1 Department of Health Sciences, School of Health & Human Sciences, Indiana University, 901 W New York St., Indianapolis, IN 46202 USA; 2 College of Nursing, Ohio State University, Columbus, OH USA; 3 Quantitative Life Sciences, McGill University, Montreal, QC Canada; 4 Department of Neurology and Neurosurgery, McGill University, Montreal, QC Canada; 5 The Montreal Neurological Institute-Hospital, McGill University, Montreal, QC Canada

**Keywords:** HF-Action, Health Belief Model, Outcomes, Heart failure, Exercise, Race

## Abstract

**Background:**

Heart Failure is a leading cause of mortality among older adults. Engaging in regular exercise at moderate-to-vigorous intensity has been shown to improve survival rates. Theory-informed methodologies have been recommended to promote exercise, but limited application of theoretical framework has been conducted for understanding racial disparities among older adults with heart failure. This study aimed to use the Health Belief Model to compare exercise behavior determinants between Black and White older adults diagnosed with heart failure.

**Methods:**

The HF-ACTION Trial is a multi-site study designed to promote exercise among individuals with heart failure that randomized participants to an experimental (three months of group exercise sessions followed by home-based training) or control arm. The present study used structural equation modeling to test the change in Health Belief Model constructs and exercise behavior across 12 months among older adults.

**Results:**

Participants (*n* = 671) were older adults, 72.28 (SD = 5.41) years old, (Black: *n* = 230; White, *n* = 441) diagnosed with heart failure and reduced ejection fraction. The model found perceived benefits, self-efficacy, perceived threats, and perceived barriers to predict exercise behavior among Black and White older adults. However, among these constructs, only perceived benefits and self-efficacy were facilitated via intervention for both races. Additionally, the intervention was effective for addressing perceived barriers to exercise only among White participants. Finally, the intervention did not result in a change of perceived threats for both races.

**Conclusions:**

Among health belief model constructs, perceived threats and barriers were not facilitated for both races in the experimental arm, and the intervention did not resolve barriers among Black older adults. Racial differences need to be considered when designing interventions for clinical populations as future studies are warranted to address barriers to exercise among Black older adults with heart failure.

Heart Failure (HF) is one of the leading causes of death among older adults in the United States [1]. Among several preventive measures, engaging in regular physical activity has consistently demonstrated to be beneficial for individuals with HF [2]. A review by Ades et al., 2013 found individuals with HF who exercised regularly demonstrated an increase in exercise capacity, decrease in clinical symptoms, improvement in quality of life, and risk reduction for future clinical events. Despite these convincing benefits, exercise participation rates among older adults with HF remains low [3]. Understanding the reasons why patients with HF forego their exercise routine can be traced to examining psychosocial factors, which have shown disparities between races. For example, at the behavioral level, there is evidence that Black individuals participate in lower levels of physical activity when controlling for social contextual factors [4] that likely contribute to exacerbating the risk of cardiac events incidence, prevalence, and recovery. At the social level, cardiac rehabilitation among Black individuals with HF is less utilized due to access barriers, lack of referral by healthcare providers, awareness or perceived benefits of cardiac rehabilitation, implicit biases, and low adherence to cardiac rehabilitation [5–7]. These findings have become salient in the literature, and hence, there have been recent calls to assess putative individual and social determinants that explain racial disparities in order to prevent and manage HF among Black individuals [8–10]. Identifying racial disparities that exist at the behavior level are best understood when employing theory-informed models that comprise behavior change determinants.

Utilizing health behavior theories in clinical populations can yield findings that are informative for improving evidence-based factors that need to be modified [11]. Moreover, applying and testing health behavior models can improve the effectiveness of interventions by increasing the comprehensive interpretation of constructs that are pivotal for promoting health behavior change. Specifically, theory-based analysis provides predictive effect sizes relative to other determinants, as all constructs in the analysis control each other’s effects. This type of analysis also allows us to rank and prioritize each theoretical construct when designing an intervention study. Among established theoretical models, the Health Belief Model (HBM) [12] can be informative for identifying racial disparities in exercise participation as it comprises several putative behavioral constructs that include perceived benefits, barriers, threats, and self-efficacy (Fig. 1). These constructs function as important intrapersonal factors for shaping healthy behaviors with each having a theoretical and conceptual interpretation [12]. For example, perceived benefits reflect the extent to which the individual believes that engaging in exercise would be healthy. Self-efficacy represents the individual’s level of confidence in performing exercise. Perceived barriers identify challenges and obstacles that would impede behavioral enactment. Finally, perceived threat assesses fears and consequences that would manifest when not performing behavior. The HBM would be insightful for identifying potential disparities in behavioral determinants between Black and White older adults when participants are placed in the same context or large randomized controlled trial, such as The Heart Failure: A Controlled Trial Investigating Outcomes of Exercise Training (HF-ACTION) [13].

**Fig. 1:**
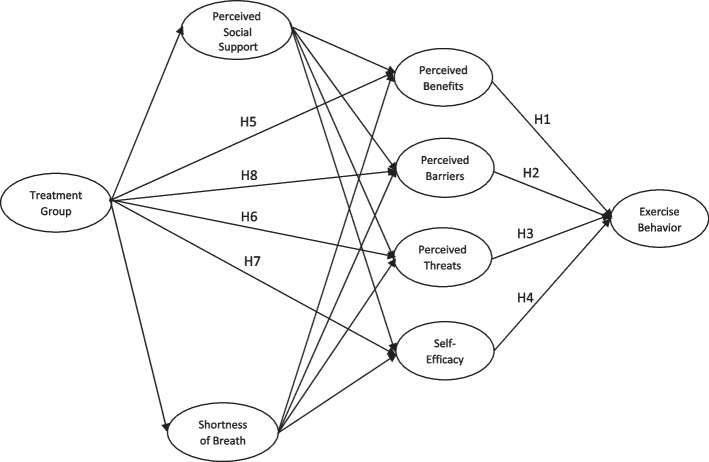
Schematic of structural equation model with hypothezied effects

The HF-ACTION trial is an international multicenter, randomized controlled trial that aimed to promote exercise among individuals who were diagnosed with left ventricular ejection fraction ≤ 35% and have heart failure symptoms for at least six weeks, based on the New York Heart Association (NYHA) class II to IV symptoms [13]. The HF-ACTION study remains one of the largest clinical trials that focused on promoting exercise training among individuals with NYHA class II to IV HF. The randomized controlled study design provides optimal data for investigating potential disparities that exist between Black and White older adult patients with HF. Hence, the purpose of this study was to use the HBM to compare exercise determinants between Black and White older adults with HF in the HF-ACTION trial with proposed hypothesized pathways depicted in Fig. 1. Specifically, it was hypothesized that HBM constructs including perceived benefits (H1), barriers (H2), threats (H3), and self-efficacy (H4) as determinants to exercise behavior would not differ between Black and White older adults with HF. It was also hypothesized that the HF-ACTION intervention would be effective in facilitating perceived benefits (H5) threats (H6), and self-efficacy (H7). However, given that the exercise program was not specifically designed for older adults, as it included all adults, we hypothesized that perceived barriers (H8) related to older adult characteristics would not be addressed for both races.

## Method

### Participants and intervention

The HF-ACTION trial is an international multi-site randomized controlled trial that randomized participants to an exercise training group or usual care. Those randomized to the exercise arm attended 3 group exercise sessions/week for three months that primarily focused on aerobic exercises. Participants fully transitioned to home-based exercise program after completing 36 sessions, where they received a treadmill or exercise bike and a heart rate monitor. Participants were instructed to exercise for 40-min sessions, five times/week, at a heart rate of 60–70%. Additional methodological details can be found in the protocol paper [13]. The study received approval from the respective Institutional Review Boards and all participants provided voluntary consent [13]. All data used from this study is stored at the National Heart, Lung, and Blood Institute and the Biologic Specimen and Data Repository (BioLINCC) accessible at https://biolincc.nhlbi.nih.gov/home and uses data from the most recent updated dataset (February 3, 2020). For the purpose of the present study, we used data of participants (*n* = 671) from the HF-ACTION trial who were older adults (age + 65 years old) and self-identified as Black or White. The investigation for the present study has been approved by the University’s research board (masked).

### Measures

The following measures, which include exercise behavior, perceived benefits, self-efficacy, perceived barriers, and perceived threats represented core model constructs that were collected during the study at baseline and at month 12. Shortness of breath and social support were collected at baseline, and these variables functioned as covariates that controlled for model effects. The HF-ACTION study was not designed to test the HBM, but the original investigators employed standardized measurements that assessed behavioral-level variables. Some of the administered scales were congruent with HBM constructs such as exercise behavior, and self-efficacy. Other model constructs that include perceived benefits, barriers, and threats were measured by selecting items from other validated scales. Reliability tests (internal consistency) was performed where applicable. Additional details are described below.

#### Exercise behavior

Exercise behavior was assessed using the International Physical Activity Questionnaire (IPAQ). The IPAQ is a validated and one of the most commonly used scales to assess physical activity behavior [14]. The present investigation focused on exercise performed both at a moderate and vigorous level, consistent with previous work on benefits of this intensity.

#### Perceived benefits

Perceived benefits were assessed using selected items from the Decisional balance scale [15]. In alignment with the definition [12] from the HBM theory, the following items were used to assess perceived benefits, “I would have more energy for my family and friends if I exercised regularly”; “Regular exercise would help me relieve tension”; “I would feel more confident if I exercised regularly”; “I would feel good about myself if I kept my commitment to exercise regularly”; “I would like my body better if I exercised regularly”; “It would be easier for me to perform routine physical tasks if I exercised regularly”; “I would feel less stressed if I exercised regularly”; “I would feel more comfortable with my body if I exercised regularly”; “Regular exercise would help me have a more positive outlook on life”. The scale demonstrated strong internal consistency (Cronbach alpha = 0.88).

#### Self-efficacy

Self-efficacy was measured using the Exercise Self-Efficacy [16]. The item was worded as follows: “The items listed below are designed to assess your beliefs in your ability to continue exercising on a three time per week basis at moderate intensities (upper end of your perceived exertion range), for 40 + minutes per session in the future”. Items were framed on a percentage Likert scale that ranged from Not at all confident (0%) to highly confident (100%). Three items were found to yield strong internal consistency (0.98), that included I am able to continue to exercise three times per week at moderate intensity, for 40 + minutes without quitting for the NEXT, i. week, ii. Two weeks, iii. three weeks.

#### Perceived barriers

Time is one of the most commonly reported barriers to exercise among older adults [17]. Time was measured using an item from the Decisional Balance Scale [15]. Participants were presented with the following question “Regular exercise would take too much of my time”, followed by 5-point Likert scale that ranged from 1 = strongly disagree to 5 = strongly agree.

#### Perceived threats

A commonly reported threat that can be improved by exercise among individuals with HF is fatigue. Supporting this finding and consistent with definition, perceived threats was assessed using items to reflect perceived susceptibility and severity of experiencing fatigue from a validated questionnaire [18] such as, “Over the past 2 weeks, how much has your fatigue bothered you?” followed by a six-point Likert scale that ranged from “extremely bothersome to I’ve had no fatigue”.

#### Shortness of breath

Shortness of breath was assessed by administering the Kansas City Cardiomyopathy Questionnaire, which is specifically designed to assess the health status of individuals with heart failure. The present study assessed items related to shortness of breath, which included, “Over the past 2 weeks, how much has your shortness of breath bothered you?”, and “Over the past 2 weeks, on average, how many times have you been forced to sleep sitting up in a chair or with at least 3 pillows to prop you up because of shortness of breath?”. Overall the questionnaire demonstrates strong psychometric properties [18]. The internal consistency for this measure was 0.79.

#### Social support

The Multidimensional Scale of Perceived Social Support is a 12-item measurement that assesses an individual’s support with regards to family, friends and significant other that are framed on a 7-point Likert scale. Examples of items include, “There is a special person who is around when I am in need”, “I can talk about my problems with my family”, and “I can count on my friends when things go wrong”. The instrument has demonstrated good test–retest reliability and sound construct validity [19] and revealed a Cronbach alpha of 0.95 for the present study.

### Analysis

Demographic data for the HF-ACTION trial has been previously reported [13]. Descriptive statistics for the model and bivariate correlations were computed using SPSS v. 28.0 [20]. The model was tested by constructing two sets of structural equation models equation modeling using IBM SPSS AMOS 22.0 [21] using full-information maximum likelihood estimation [22]. The model included pathways from each HBM construct to MVPA (exercise behavior) to identify if there were between race differences in the requirements to engage in exercise. This was followed by extending pathways from the treatment group variable (experimental vs. control) to each HBM determinant, to identify if there were racial discrepancies in the development of each HBM construct. Visualization of effect sizes in the model were depicted as a Sankey diagram using the Sankey package [23] and Matplotlib plotting library for Phyton [24]. Given that our hypotheses tested change in HBM constructs across 12 months, change (or residual) scores for model items were computed by regressing each item value at 12 months on its baseline value, which is a well-documented approach to computing model change [25–27]. The model controlled for shortness of breath, which is a commonly reported symptom among HF patients when exercising, and their perceived social support, which could influence their participation levels. Group analysis feature (Black vs. White) was used to test pathways for each of the two groups in AMOS. Model fit indices were assessed by commonly reported parameters that include Hu and Bentler’s two-index presentation strategy (1999), which states that the standardized root mean square residual (SRMR) value should be less than 0.09 and that the comparative fit index (CFI) should be greater than or equal to 0.90 [28]. Model fit was also assessed by observing an additional set of recommended parameters [29], which included Tucker-Lewis Index (TLI) having value greater than 0.95 for strong fit [30] and root mean square error of approximation (RMSEA) value lower than 0.05 for strong, or less than 0.08 for acceptable fit [30]. Path effect sizes were reported using standardized beta coefficients.

## Results

Data was included from Black and White older adults (age 65 +) who had completed data, which of the 2,331 participants yielded 230 Black and 441 White individuals. Participants were 72.28 (SD = 5.41) years old and reported engaging in an average of 169 min (*SD* = 141) of moderate-to-vigorous exercise per week at baseline. Black (M = -0.005, SD = 0.57) and White (M = -0.073, SD = 0.60) participants demonstrated a decline in MVPA at 12-month mark. Baseline characteristics of the cohort by race (White / Black) can be found in Table 1. Correlations supported HBM theorizing as behavior was found to correlate with perceived beliefs, (r = 0.59, *p* < 0.001), threats (r = 0.54, *p* < 0.001), self-efficacy (r = 0.19, *p* < 0.001), and barriers (r = 0.46, *p* < 0.001). Additional correlations can be found in Table 2.

**Table 1:** Baseline characteristics of the cohort by race (White / Black)

Variables	**White (*n* = 441)**	**Black (*n* = 230)**
Age (years)	72.34 ± 5.32	72.16 ± 5.58
Male (%)	341 (77.3%)	182 (79.1%)
**Education**		
Less than high school	74 (16.8%)	23 (10.0%)
High school graduate or equivalent	107 (24.3%)	63 (27.4%)
Completed some college, but no degree	93 (21.1%)	62 (27%)
Completed associate degree/diploma program	26 (5.9%)	16 (7.0%)
College graduate	79 (17.9%)	36 (15.7%)
Completed graduate school	48 (10.9%)	28 (12.2%)
No answer	14 (3.2%)	2 (0.9%)
**Income**		
< $15,000	61 (13.8%)	29 (12.6%)
$15,000 – $24,999	86 (19.5%)	48 (20.9%)
$25,000 – $ 34,999	62 (14.1%)	36 (15.7%)
$35,000—$49,999	65 (14.7%)	35 (15.2%)
$50,000—$74,999	59 (13.4%)	34 (14.8%)
$75,000—$99,000	21 (4.8%)	16 (7%)
> $100,000	25 (5.7%)	13 (5.7%)
No answer	62 (14.1%)	19 (8.3%)
**Clinical Characteristics**		
Resting Heart Rate (bpm)	68.16 ± 9.90	68.16 ± 9.89
SBP (mmHg)	117.40 ± 18.63	117.48 ± 17.98
DBP (mmHg)	68.86 ± 10.67	68.85 ± 10.72
PP (mmHg)	48.54 ± 14.82	48.70 ± 13.90
LVEF (%)	25.92 ± 7.60	25.36 ± 7.46
BNP (pg/mL)	596.28 ± 743.28	563.75 ± 638.38
Pro-BNP (pg/mL)	4894.64 ± 7219.58	6100.33 ± 8278.20
BMI (Kg/m^2^)	28.08 ± 5.38	29.07 ± 5.17
**New York Heart Association Classification of Heart Failure (%)**		
Class II	267 (60.5%)	138 (60.0%)
Class III	168 (38.1%)	88 (38.3%)
Class IV	6 (1.4%)	4 (1.7%)

*SBP* Systolic Blood Pressure, *DBP* Diastolic Blood Pressure, *BMI* Body Mass Index, *PP* Pulse Pressure, *LVEF* Left Ventricular Ejection Fraction, *BNP* B-type natriuretic peptide

**Table 2:** Bivariate correlations among study variables

Construct	1	2	3	4	5
1. Beliefs	1.00	.24***	.48***	.47***	.59***
2. Self-Efficacy		1.00	.52***	.38***	.19**
3. Perceived Barriers			1.00	.58***	.46***
4. Perceived Threats				1.00	.54***
5. Exercise Time					.100

Coefficients presented are zero-order bivariate correlations at baseline

Behavior Exercise at moderate-to-vigorous intensity

^*^ p < .05

^**^ p < .01

^***^ p < .001

### Model fit and hypothesis tests

The SEM model displayed strong model stability (**χ** ^2^ = 2621, df = 77, *p* < 0.001; RMSEA = 0.05 (95% CI 0.04, 0.064); CFI = 0.96; TLI = 0.95; SRMR = 0.05). Factor loadings for model constructs can be found in Table 3. The findings report behavioral determinants between each race (H_1_—H_4_), followed by if the intervention group facilitated the determinants between each race (H_5_-H_8_).

**Table 3:** Means, standard deviations and factor loadings of model items

Indicator	Factor Loadings
1. Benefits.1	.83
2. Benefits.2	.81
3. Benefits.3	.64
4. Self-Efficacy.1	.82
5. Self-Efficacy.2	.84
6. Self-Efficacy.3	.82
7. Self-Efficacy.4	.73
8. Self-Efficacy.5	.72
9. Perceived Threat.1	.74
10. Perceived Threat.2	.72
11. Perceived Barriers^a^	
12. Behaivor^a^	

^a^Construct measured using a single item, so no factor loading reported; Perceived Behavioral Control

### Model tests

**Fig. 2:**
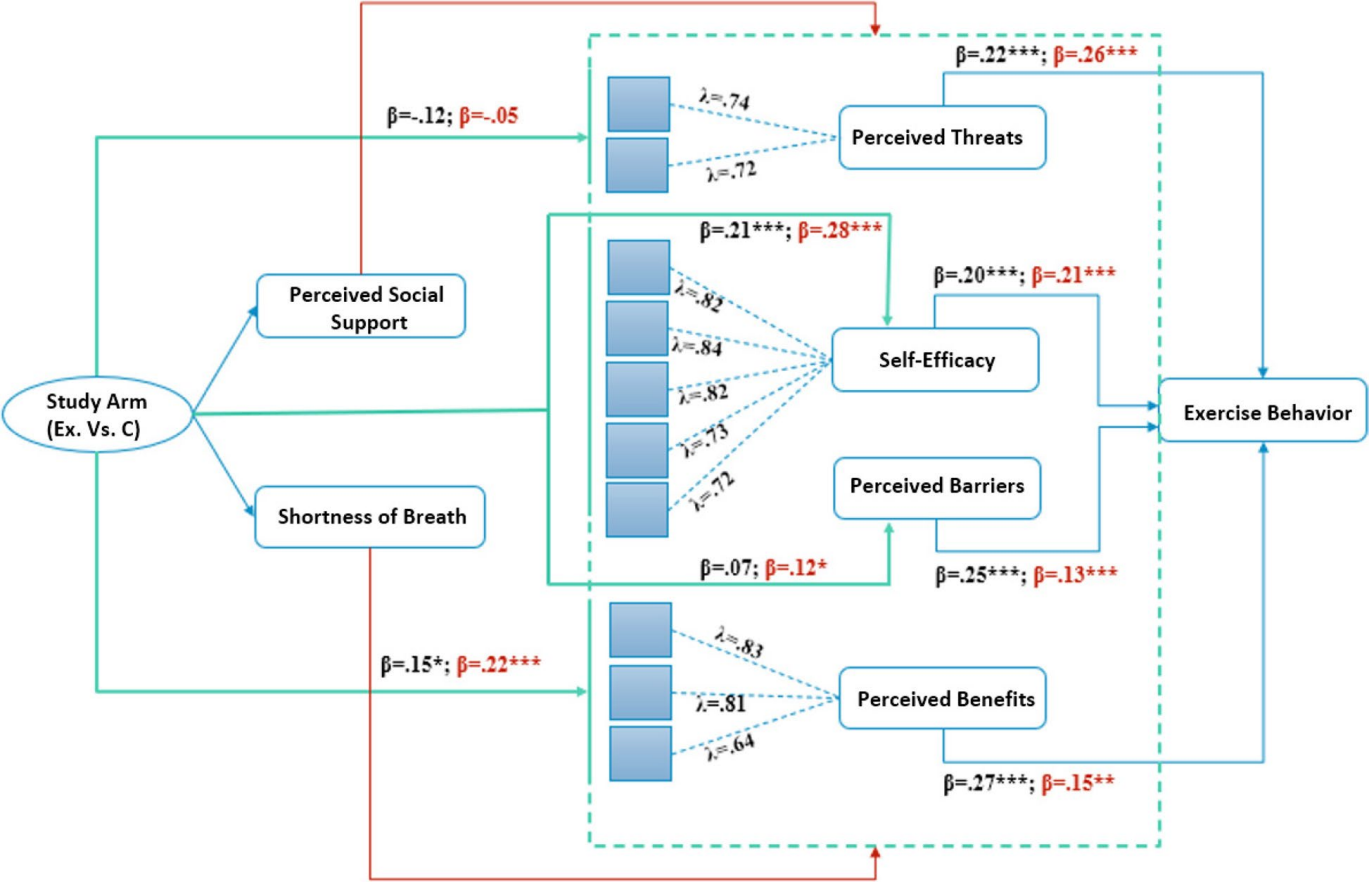
Structural Equation model with Tested Effects The figure presents the pathways from structural equation modeling that investigated the relationship between health belief model constructs among participants. Study Arm variable represents experimental vs. control arm. All values are standardized regression coefficients. Black font coefficients represent Black participants and red font coefficients represent White participants. Green pathways show the the prediciton from study arms to each health belief model construct. Blue pathways show effects from health belief model constructs to exercise behavior. Red pathways are controlled effects. *p* < .01, **, *p* < .001***

**Fig. 3:**
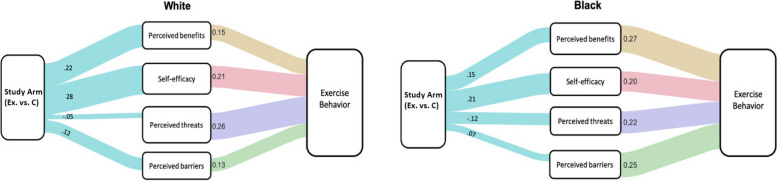
Sankey Diagram The Sankey figure displays the model effects for each race for comparison. The model included pathways from each Health Belief Model (HBM) construct to exercise behavior to identify racial differences in exercise determinants. Path A shows HBM determinants predicting exercise behaviors in racial groups. Path B indicates how study arm (experimental vs control) facilitate each HBM determinant. All values represent standardized regression coefficients

The tested structural equation model can be found in Fig. 2, and the Sankey model can be found in Fig. 3. Consistent with the hypotheses H_1_ -H_4_, HBM determinants including perceived benefits (Black: β = 0.27, *p* < 0.001; White: β = 0.15; *p* = 0.003), self-efficacy (Black: β = 0.20, *p* = 0.003; White: β = 0.21; *p* < 0.001), perceived threats, (Black: β = 0.22, *p* = 0.004; White: β = 0.26; *p* < . 001), and barriers (Black: β = 0.25, *p* < 0.001; White β = 0.13; *p* = 0.003) were found to be significant determinants among Black and White older adults. Hypotheses H_5_ -H_8_ tested if intervention facilitated these determinants in both races. Study arm found the experimental arm to be effective in predicting perceived benefits (Black: β = 0.15. *p* = 0.037; White: β = 0.22; *p* < . 001) and self-efficacy (Black: β = 0.21, *p* < 0.001; White: β = 0.28; *p* < 0.001) for both races. However, the intervention was effective for informing perceived time barrier among White (β = 0.12; *p* = 0.010) but not Black participants (Black: β = 0.07, *p* = 0.263). Finally, the intervention did not address perceived threats for both races (Black: β = -0.12; *p* = 0.054; White: β = -0.05, *p* = 0.211).

## Discussion

Heart failure is a cardiovascular disease that disproportionately affects older adults, and is found to have more severe outcomes among Black individuals [1]. There is growing evidence to support that Black patients with HF experience health inequities in healthcare settings such as shorter physician times, misdiagnosis, suggesting lower consideration in treatments [8]. The present study investigated if inequitable patterns for promoting exercise also extend to older adult Black patients with HF in a large clinical trial. The primary objective tested a set of hypotheses to identify disparities in HBM constructs to predict exercise participation. The findings revealed perceived benefits, threats, barriers, and self-efficacy predicted exercise behavior for Black and White older adults, indicating that racial groups likely require the same behavioral determinants to participate in exercise. The secondary objective tested if the HF-ACTION intervention provided equitable support for enhancing HBM behavioral determinants. The findings indicated that the exercise program was effective in facilitating self-efficacy and perceived benefits of exercise for both racial groups. However, the intervention was not effective in assisting Black patients with their perceived time barriers to exercise. Additionally, the HF-ACTION intervention did not improve perceived threats for both races.

Supporting the HBM model [12], proposed determinants including perceived benefits, threats, barriers, and self-efficacy were found to predict exercise behavior for both races, which is consistent with the original theoretical pathways [31, 32]. The intervention was demonstrated to be effective for fostering positive attitudes, and improving self-efficacy for both races, but the intervention showed racial differences in minimizing perceived barriers. Time is one of the most commonly documented barriers to exercise for mid-age and older adults [17], which was also shown to be predictive of exercise behavior in the present study, but was not resolved among Black patients by the exercise intervention. Social and personal factors such as family dynamics/ responsibilities might need to be considered when designing interventions for different racial groups to understand time allocation and management. Then, behavior-regulation strategies such as planning, and habit formation techniques, which are effective for securing protected time to exercise [33–38], can be facilitated in an education program. Specifically, experimental research has found clinical populations, such as those in cardiac rehabilitation such as acute coronary syndrome, are able to apply these behavior change techniques [39, 40].

The findings from the tested Health Belief Model (HBM) also revealed that while perceived threats were predictive factor for exercise participation, this construct may not have been addressed in the intervention group. Perceived threats are a behavior level construct that has been shown to be associated with several health behaviors. Specifically, previous work supports the associations between physical activity and perceived threats among older adults [41]. Other related findings has shown the severity/seriousness component of threat construct to predict intentions to exercise among patients with coronary artery disease [42]. Overall, facilitating HBM determinants for patients with HF to exercise regularly via education-focused intervention needs to be tested, as education-based interventions based on this model have been demonstrated to be effective for patients with other cardiovascular diseases [43, 44].

### Strengths, limitations, and future directions

To the best of our knowledge, this is the first study to investigate exercise behavioral determinants among individuals with HF using the HBM. This is also the largest investigation to compare differences in theoretical determinants of exercise behavior between racial groups in the cardiovascular-related literature. The findings address emerging calls to identify the impact of psychosocial factors in explaining the racial disparities in exercise behaviors among Black patients with HF [8–10]. While these results demonstrate similar and distinct patterns between Black and White older adults in the HF ACTION trial, it is important to note that the intervention was not designed for older adults. It is also worth noting that the HBM proposes “cues to action” to predict behavioral enactment, though we could not find a relevant variable in the dataset to reflect this construct. Cues have been shown to have mixed findings and consequently have been omitted in several studies [31], though cues have also been found to be important [45, 46] and in most cases its omission can be attributed to complexity of precisely assessing this measurement with habit [47]. This note leads to an important future direction for large scale clinical trials related to cardiovascular disease to include dedicated measures related to a behavior theory to yield robust findings.

## Conclusion

The Health Belief Model revealed to be effective for understanding exercise behavior among older adults with HF. Augmenting the HBM to include self-regulation strategies such as planning, and habit formation might be effective for addressing time-related barriers. Overall, the importance of this investigation provides two key notes for future studies. The first is the significance of an exit study interview to welcome insight that might not be captured via standardized quantitative measures. For instance, in this study, it is not clear why Black individuals perceived greater time barrier to exercise participation compared to their White counterparts. Second, these discrepant findings were revealed using theory-informed analyses. Future clinical trials that include HF patients should be planned to measure and deliver an intervention based on an established theoretical framework. Translating these findings into real world applications signify the importance of considering behavioral, environmental, and biological (age, race) characteristics of individuals with HF to determine the feasibility exercise prescriptions by healthcare providers. Additionally, partnerships between clinics and community organizations such as recreation centers can serve as pivotal factors in helping HF patients establish and maintain a consistent exercise routine. This is especially important in Black communities where distinct behavioral and environmental barriers might need to be addressed, which necessitates a tailored approach for promoting exercise.

## Data Availability

Data used in this study can be found here: https://biolincc.nhlbi.nih.gov/home

## References

[1] Sidney S (2019). Association Between Aging of the US Population and Heart Disease Mortality From 2011 to 2017. JAMA Cardiology.

[2] Ades PA (2013). Cardiac rehabilitation exercise and self-care for chronic heart failure. JACC Heart Fail.

[3] Flint KM, Forman DE (2018). Lessons From the First 202 REHAB-HF Participants. Circ Heart Fail.

[4] Wilson-Frederick SM (2014). Examination of race disparities in physical inactivity among adults of similar social context. Ethn Dis.

[5] Mathews L (2022). Disparities in the Use of Cardiac Rehabilitation in African Americans. Curr Cardiovasc Risk Rep.

[6] Koehler Hildebrandt AN (2016). Biopsychosocial-spiritual factors impacting referral to and participation in cardiac rehabilitation for african american patients. J Cardiopulm Rehabil Prev.

[7] Lewsey SC, Breathett K (2021). Racial and ethnic disparities in heart failure: current state and future directions. Curr Opin Cardiol.

[8] Nayak A, Hicks AJ, Morris AA (2020). Understanding the Complexity of Heart Failure Risk and Treatment in Black Patients. Circ Heart Fail.

[9] White-Williams C (2020). Addressing Social Determinants of Health in the Care of Patients With Heart Failure: A Scientific Statement From the American Heart Association. Circulation.

[10] Youmans QR, Lloyd-Jones DM, Khan SS (2022). Race, Ancestry, and Risk: Targeting Prevention to Address Heart Failure Disparities. Circ Heart Fail.

[11] Glanz, K. and D.B. Bishop, The role of behavioral science theory in development and implementation of public health interventions, in Annual Review of Public Health. 2010. p. 399–418. 10.1146/annurev.publhealth.012809.103604 20070207

[12] Rosenstock IM (1974). The Health Belief Model and Preventive Health Behavior. Health Educ Monogr.

[13] O'Connor CM (2009). Efficacy and safety of exercise training in patients with chronic heart failure: HF-ACTION randomized controlled trial. JAMA.

[14] Craig CL (2003). International physical activity questionnaire: 12-country reliability and validity. Med Sci Sports Exerc.

[15] Marcus BH, Rakowski W, Rossi JS (1992). Assessing motivational readiness and decision making for exercise. Health Psychol.

[16] McAuley E, Lox C, Duncan TE (1993). Long-term maintenance of exercise, self-efficacy, and physiological change in older adults. J Gerontol.

[17] Justine M (2013). Barriers to participation in physical activity and exercise among middle-aged and elderly individuals. Singapore Med J.

[18] Green CP (2000). Development and evaluation of the Kansas City Cardiomyopathy Questionnaire: a new health status measure for heart failure. J Am Coll Cardiol.

[19] Zimet GD (1988). The Multidimensional Scale of Perceived Social Support. J Pers Assess.

[20] IBM, IBM SPSS Statistics for Windows, Version 28.0, ed. C.S.R.S. Inc. 2021, Armonk, NY: IBM Corp.

[21] Arbuckle, J.L., Amos (Version 22.0) 2014: IBM SPSS

[22] Enders CK (2011). Analyzing longitudinal data with missing values. Rehabil Psychol.

[23] Golob, A., M. Manz, and P. Sassoulas. Normalized Sankey Diagrams. [cited 2022; Available from: https://github.com/anazalea/pySankey.

[24] Hunter, J.D., Matplotlib: A 2D Graphics Environment. Computing in Science & Engineering, 2007. 9(3).

[25] Buffart LM (2014). Mediators of the resistance and aerobic exercise intervention effect on physical and general health in men undergoing androgen deprivation therapy for prostate cancer. Cancer.

[26] Kaushal N (2017). The role of habit in different phases of exercise. Br J Health Psychol.

[27] Arnautovska U (2017). A longitudinal investigation of older adults’ physical activity: Testing an integrated dual-process model. Psychol Health.

[28] Hu LT, Bentler PM (1999). Cutoff criteria for fit indexes in covariance structure analysis: Conventional criteria versus new alternatives. Struct Equ Model.

[29] Arbuckle, J.L., Amos 20 User's Guide. Chicago, IL.: SPSS Inc. 2011.

[30] Byrne, B., Structural Equation Modeling With AMOS: Basic Concepts, Applications, and Programming, Third Edition (Multivariate Applications Series). Multivariate Applications Series. 2016, London, UK: Routledge.

[31] Jones CJ, Smith H, Llewellyn C (2014). Evaluating the effectiveness of health belief model interventions in improving adherence: a systematic review. Health Psychol Rev.

[32] Sulat JS (2018). The validity of health belief model variables in predicting behavioral change: A scoping review. Health Educ.

[33] Kaushal N (2020). Social Cognition and Socioecological Predictors of Home-Based Physical Activity Intentions, Planning, and Habits during the COVID-19 Pandemic. Behav Sci.

[34] Kaushal N (2018). Mediating Mechanisms in a Physical Activity Intervention: A Test of Habit Formation. J Sport Exerc Psychol.

[35] Kaushal, N., et al., Increasing Physical Activity Through Principles of Habit Formation in New Gym Members: a Randomized Controlled Trial. Annals of Behavioral Medicine, 2017: p. 1–9. 10.1007/s12160-017-9881-5 28188586

[36] Koh LH (2017). Effects of a brief action and coping planning intervention on completion of preventive exercises prescribed by a physiotherapist among people with knee pain. J Sci Med Sport.

[37] Fournier M, d'Arripe-Longueville F, Radel R (2017). Testing the effect of text messaging cues to promote physical activity habits: a worksite-based exploratory intervention. Scand J Med Sci Sports.

[38] Fournier M (2017). Effects of circadian cortisol on the development of a health habit. Health Psychol.

[39] Kaushal N (2022). How and Why Patients Adhere to a Prescribed Cardiac Rehabilitation Program: A Longitudinal Phenomenological Study of Patients with Acute Coronary Syndrome. Int J Environ Res Public Health.

[40] Kaushal N (2021). Facilitating Exercise Habit Formation among Cardiac Rehabilitation Patients: A Randomized Controlled Pilot Trial. Int J Environ Res Public Health.

[41] Preissner CE (2023). A Protection Motivation Theory Approach to Understanding How Fear of Falling Affects Physical Activity Determinants in Older Adults. J Gerontol B Psychol Sci Soc Sci.

[42] Tulloch H (2009). Predicting short and long-term exercise intentions and behaviour in patients with coronary artery disease: A test of protection motivation theory. Psychol Health.

[43] Saffari, M., et al., An Intervention Program Using the Health Belief Model to Modify Lifestyle in Coronary Heart Disease: Randomized Controlled Trial. Int J Behav Med, 2023. 10.1007/s12529-023-10201-1 37477851

[44] Habibzadeh H (2021). The effect of patient education based on health belief model on hospital readmission preventive behaviors and readmission rate in patients with a primary diagnosis of acute coronary syndrome: a quasi-experimental study. BMC Cardiovasc Disord.

[45] Lilly FRW (2020). Pathways from health beliefs to treatment utilization for severe depression. Brain and Behavior.

[46] Spinewine A (2021). Attitudes towards COVID-19 Vaccination among Hospital Staff-Understanding What Matters to Hesitant People. Vaccines.

[47] Kaushal N (2021). Differences and similarities of physical activity determinants between older adults who have and have not experienced a fall: Testing an extended health belief model. Arch Gerontol Geriatr.

